# Subcutaneous Emphysema of Periorbital and Canine Space Following Endodontic Treatment

**DOI:** 10.7759/cureus.56307

**Published:** 2024-03-17

**Authors:** Kamalakannan Padmanaban, Viswanathan Revathy, Davidson Rajiah, Priyadharshini Raghavan

**Affiliations:** 1 Department of Oral and Maxillofacial Surgery, Tamilnadu Government Dental College and Hospital, Chennai, IND; 2 Department of Pediatric Dentistry, Tamilnadu Government Dental College and Hospital, Chennai, IND

**Keywords:** soft tissue crepitus, endodontic treatment, canine space swelling, periorbital swelling, subcutaneous emphysema

## Abstract

Emphysema of the subcutaneous tissue is an uncommon complication of dental procedures. Certain dental surgical procedures, such as extraction of teeth using air-driven handpieces and endodontic procedures are more prone to cause subcutaneous emphysema. Subcutaneous emphysema is typically self-limiting and only in a few instances has an impact on the long-term health of the patient. Patients with subcutaneous emphysema experience pain, distress and inconvenience. This paper presents a case of subcutaneous emphysema of the right canine and periorbital space following endodontic treatment of the upper right front tooth.

## Introduction

The Greek term "emphusan," which means "puff up," is from where the word "emphysema" originates. Subcutaneous emphysema, also known as tissue emphysema, develops when gas or air is entrapped in the subcutaneous layer. Emphysema caused by a surgical procedure is referred to as surgical emphysema [1,2]. During surgical removal of teeth or during the provision of restorative therapy, including direct restorations or while preparing the teeth for crowns, the use of high-speed dental handpieces that generate air has been documented to be associated with the development of subcutaneous emphysema [3]. It is a potential side effect of both surgical and nonsurgical endodontic therapy. The most frequent causes are excessive hydrogen peroxide irrigation or compressed air blasts used to dry the root canals [1]. Patients with preexisting periodontal disease are at a higher risk of developing subcutaneous emphysema [4].

The primary mechanism by which subcutaneous emphysema develops is through the forced entry of pressured air into fascial planes via the periodontal tissues through a perforation site or a large apical root foramen [1]. When the upper teeth are involved, canine space and periorbital swelling are more likely to occur, and when the mandibular teeth are involved, cheek and neck swellings are the most frequent [5]. Larger volumes of air can sometimes move into deeper fascial planes [1].

The purpose of this article is to describe a case of subcutaneous emphysema following endodontic treatment in an eight-year-old and discuss the possible etiologies and clinical symptoms of subcutaneous emphysema. It also aims to emphasize the diagnosis, management, and clinical guidelines for the prevention of subcutaneous emphysema during dental procedures.

## Case presentation

An eight-year-old patient was referred to the Department of Oral and Maxillofacial Surgery from the Department of Pedodontics with complaints of swelling in the right cheek and periorbital region that developed spontaneously while an endodontic procedure was being performed. The patient had reported to the Department of Pedodontics for management of a carious upper right deciduous canine, for which an endodontic procedure was carried out following administration of local anesthetic (2cc of 2% lignocaine with 1:80000 adrenaline). During the procedure, the patient developed an acute swelling in the right canine fossa region, which extended into the periorbital area.

**Figure 1:**
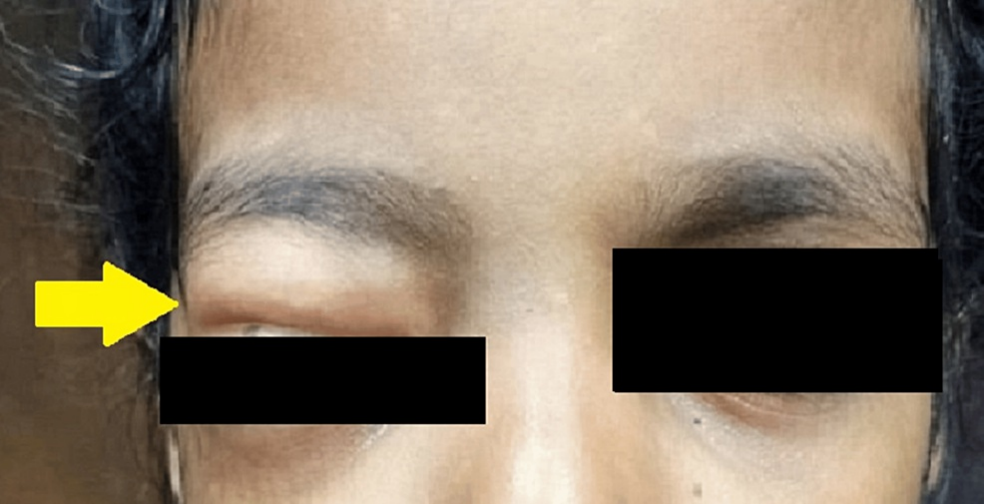
Swelling over the right periorbital region

**Figure 2:**
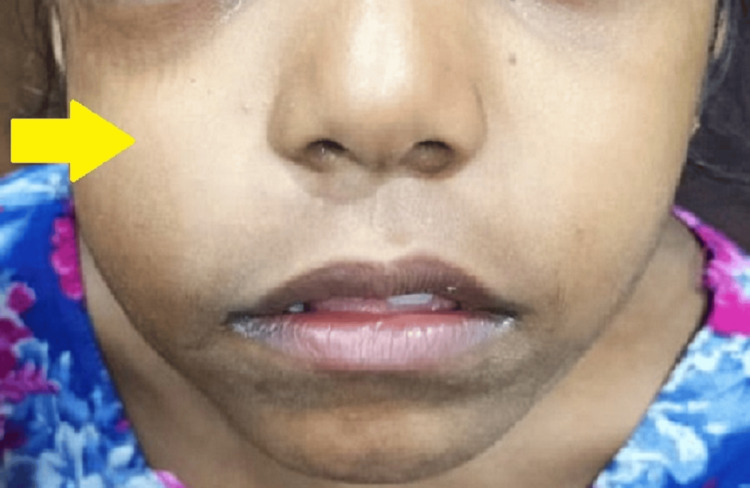
Swelling over right canine fossa region

Examination revealed facial asymmetry caused by diffuse, soft, and painless unilateral swelling with obvious crepitus in the right periorbital and maxillary regions (Figures 1, 2). Examination of cranial nerves I - VII revealed mild restriction of movement in the right eye. The patient was administered injection dexamethasone 4 milligrams intravenously as the initial line of management, following which she was kept under observation. The patient was referred to the Institute of Ophthalmology for further ophthalmological examination and intervention if needed. On ophthalmic examination, there was right periorbital edema, mechanical ptosis of the right eye, and a mild restriction of elevation in the right eye. Visual acuity, direct and indirect pupillary light reflexes were normal. The patient was prescribed antihistamines and antibiotics (tablet chlorpheniramine 2 milligrams four times a day, tablet amoxicillin + potassium clavulanate 375 milligrams twice a day and tablet metronidazole 200 milligrams four times a day) for five days.

The differential diagnosis made based on the clinical findings were subcutaneous emphysema, allergic reaction, hematoma, and angioneurotic oedema [1]. Allergic reactions are far more severe than subcutaneous emphysema, with skin manifestations preceding serious cardiorespiratory manifestations, and the associated swelling is typically firmer in consistency and presents with other local features like increased erythema and urticaria [1]. In our case, the swelling was localized to the face with no systemic reactions, thus ruling out the possibility of an allergic reaction. As far as hematoma is concerned, formation is rapid and often without initial discoloration. Although sponginess may be present, crepitus is absent in hematomas [1]. Hence, the presence of crepitus ruled out the possibility of hematoma. In angioneurotic edema, circumscribed areas of edema, sometimes preceded by a burning sensation, may appear on the skin or mucous membrane [1]. It usually presents with a swelling of the lips, which was absent in this case.

**Figure 3:**
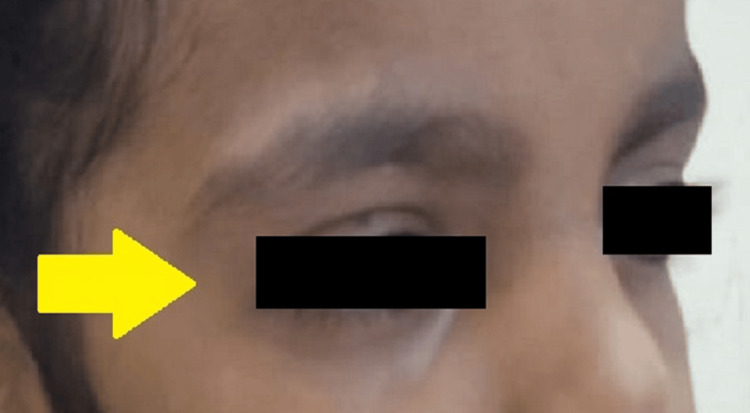
Three days following the incidence of the swelling showing complete resolution near right periorbital region

**Figure 4:**
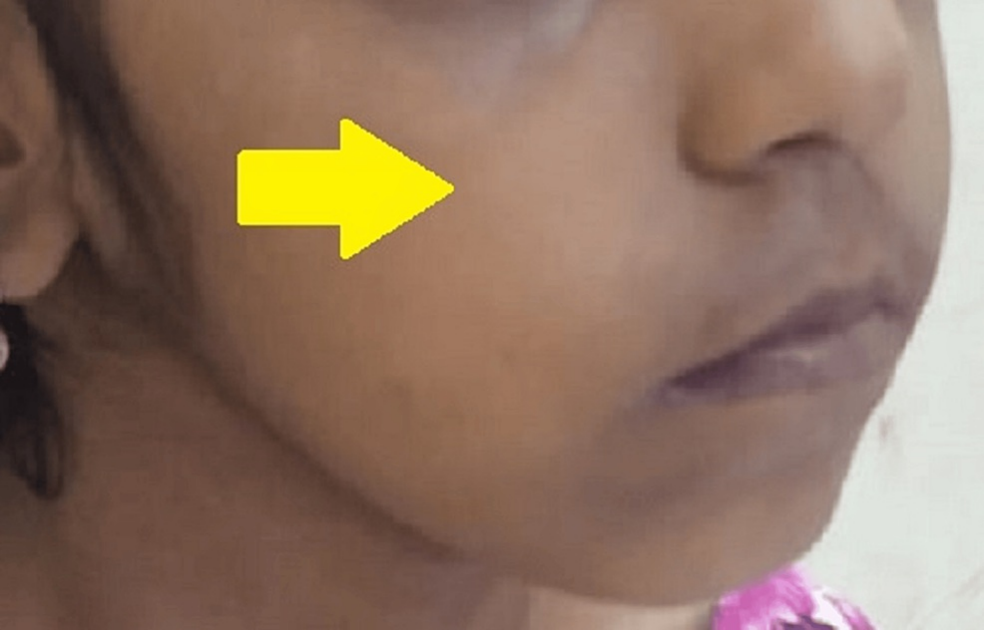
Three days following the incidence of the swelling showing complete resolution near right canine fossa region

A final diagnosis of subcutaneous emphysema was made based on the patient's history and clinical findings. The patient was followed up regularly, and complete resolution was observed within three days (Figures 3, 4).

## Discussion

Turnbull reported the first account of subcutaneous emphysema associated with premolar extraction in 1900, when a musician blew a trumpet shortly after having a tooth extracted [6,7]. The use of high-speed air or water drills during dental procedures has been linked to subcutaneous emphysema. The disruption of the dento-alveolar membrane or perforation of the root canal during tooth extraction, restorative dentistry, dental implant surgery, root canal treatment, or periodontal treatment can result in the invasion of compressed air into soft tissues. The intraoral barrier is compromised during all of these procedures, allowing air under pressure to travel beneath the skin. During standard root canal therapy, hydrogen peroxide has been used as an irrigant and a disinfectant. When hydrogen peroxide comes into contact with the proteins in blood or tissue, it quickly goes through an effervescence process and releases oxygen. If the canal wall was accidentally perforated, this gaseous expansion may push gas or debris into the neighboring bone or into the apical foramen [1].

In our case, the air entrapment in the canine and the periobital space could have been caused by air blown through a three-way syringe into the root canal and the adjacent socket of the exfoliated upper right deciduous lateral incisor, which later provided a passage for the seepage of local anesthetic agent into the periorbital space, causing mild diplopia.

Rapid swelling of the face and even the neck is the most noticeable clinical symptom of subcutaneous emphysema. The affected area swells up, and palpation always reveals the presence of crepitus. Treatment for mild to moderate cases consists of patient observation and reassurance [1]. Emphysema typically resolves in two to three days [3]. It is advised to gently irrigate the region with distilled water through the portal of entrance if substances like hydrogen peroxide (H2O2) or sodium hypochlorite (NaOCl) are suspected to be the cause of subcutaneous emphysema. Since the introduction of air from the oral cavity usually drives microorganisms along with it, broad-spectrum antibiotics are usually prescribed. In severe cases, prompt medical care is essential [1].

Subcutaneous emphysema can be avoided by using a well-fitted rubber dam, electric motor-driven handpieces, high-speed aspiration, or paper points to dry fluids from the root canal during dental procedures [1].

## Conclusions

To conclude, subcutaneous emphysema resulting from dental treatments is uncommon but can have a potentially fatal consequence if not identified and treated promptly. Less severe cases usually require observation, reassurance and prescription of broad-spectrum antibiotics so as to reduce the possibility of infection while severe cases require prompt medical care. Usage of rubber dam, electric motor-driven handpieces, high-speed aspiration, or paper points to dry fluids from the root canal are a few ways to prevent the occurrence of subcutaneous emphysema during dental procedures.
